# Identifying triggers for optimal timing of advance care planning in electronic primary health care records: a nested case-control study

**DOI:** 10.1136/bmjopen-2025-104742

**Published:** 2025-11-29

**Authors:** Willemijn Tros, Jenny van der Steen, Mattijs E Numans, Marta Fiocco, Petra G van Peet

**Affiliations:** 1 Public Health and Primary Care, Leiden University Medical Center, Leiden, The Netherlands; 2 Primary and Community Care and Radboudumc Alzheimer Center, Radboud University Medical Center, Nijmegen, The Netherlands; 3 Cicely Saunders Institute, King’s College London, London, UK; 4 Mathematical Institute, Leiden University, Leiden, The Netherlands; 5 Medical Statistics Department of Biomedical Data Science, Leiden University Medical Center, Leiden, The Netherlands; 6 Prinses Maxima Centrum voor Kinderoncologie BV, Utrecht, The Netherlands

**Keywords:** PALLIATIVE CARE, Clinical Decision-Making, Electronic Health Records, Frail Elderly, Primary Care, Aged

## Abstract

**Abstract:**

**Objectives:**

To explore whether routine electronic healthcare records can be used to identify triggers for initiating advance care planning (ACP) and the optimal time window to initiate ACP. We aimed to assess the prevalence of triggers for initiating ACP as defined for use in routine data, whether their presence is associated with death, and what their position is relative to a previously identified ‘optimal time window for ACP’.

**Design:**

Nested case-control study within a large dynamic population cohort dataset.

**Setting:**

Primary care population-based, anonymised data extracted from GP centres in the South Holland province, The Netherlands.

**Participants:**

We selected records of individuals aged ≥65 registered with their general practice from 1 Jan 2014 to 1 Jan 2017. Cases were individuals who died between 1 Jan 2017 and 1 Jan 2020. Controls were individuals who remained alive. Cases were matched by age to controls in a 1:4 ratio.

**Main outcome measures:**

Outcomes include prevalence of triggers for ACP in the records of deceased and living individuals; association of the triggers’ presence with death; timing of the identified triggers in deceased individuals relative to the ‘optimal time window for ACP’.

**Results:**

We included 17098 records, 4139 from deceased individuals (mean age 81) and 12959 from living individuals (mean age 79). Triggers most strongly associated with death were consultations concerning malignancy (OR 8.35, 95% CI 7.42 to 9.41), hospital admissions (OR 7.32, 95% CI 6.75 to 7.94), emergency department referrals (OR 7.11, 95% CI 6.52 to 7.75), registered home visits (OR 5.97, 95% CI 5.51 to 6.47), consultations concerning heart failure (OR 5.25, 95% CI 4.59 to 5.99), dementia (OR 4.75, 95% CI 3.99 to 6.56), opioid prescriptions (OR 4.58 (4.25–4.93), consultations concerning general decline/feeling old (OR 4.15, 95% CI 3.72 to 4.64) and skin ulcers/pressure sores (OR 4.04, 95% CI 3.55 to 4.61). Those closest to the median of the optimal time window for ACP were consultations regarding dyspnoea, general decline/feeling old, heart failure, skin ulcers/pressure sores and fever, opioid prescriptions, emergency department referrals, registered home visits and hospital admissions.

**Conclusions:**

Clinical triggers for initiating ACP in general practice can be recognised within the routine electronic health records and they align well with the ‘window of opportunity’ to initiate ACP.

**STRENGTHS AND LIMITATIONS OF THIS STUDY**

- The study’s focus on triggers for initiating ACP across an entire general practice population, rather than triggers for imminent death or in specific diseases, results in the identification of a broader and more generalisable range of potentially valuable triggers.
- The study used real-life data ensuring applicability in clinical practice.
- The pragmatic choice of using end of life as a reference in some of our analyses does not align with the view that ACP should not be limited to end-of-life communication.
- The study incorporated national data in the analysis, though such data may not always be readily obtained in general practice settings.
- The study population was relatively old due to matching with deceased individuals, which may limit generalisability to other populations aged 65 and over.

## Introduction

 As people live longer with more chronic conditions, healthcare should focus, besides on prolonging life, on quality of life and palliative care.^1^,^3^ The significance of advance care planning (ACP) in this context is increasingly acknowledged.^4^,^6^ ACP is a process which enables patients to specify and share their values, goals and preferences for future medical care,^7^ ideally embedded in primary care, closely tied to home.^8 9^ However, ACP remains inconsistently implemented in general practice and, if performed, often occurs late in the disease trajectory, particularly for patients with non-malignant diseases.^10^,^13^ This may be due to the challenges in timing ACP, and guidance for healthcare professionals on appropriate timing is needed.^8 14^

Although tools exist to identify patients requiring palliative care or nearing the end of life, potentially guiding healthcare professionals to initiate ACP, these are underutilised due to time constraints and lack of integration into routine practice.^15 16^ Electronic health records (EHR), incorporating, for instance, automated screening algorithms and built-in reminders, are promising tools for identifying patients who could benefit from initiation of ACP.^17^,^19^ However, findings on their efficacy are mixed^20 21^ and the research focuses on end-of-life identification. ACP, however, should not be confined to the end of life; it is already relevant for individuals earlier in the course of chronic-progressive disease. Our previous study identified optimal timing as a ‘window of opportunity’, which is broader than a single optimal point and consistent with a period of preferred timing indicated by GPs. Further, we identified triggers for ACP initiation such as ‘receiving a diagnosis of a life-threatening or a chronic-progressive illness’, ‘after a period of sickness’, ‘functional deterioration’, ‘acute symptoms’ and ‘general deterioration’.^22^

Our study aims to explore if routine electronic healthcare records can be used to identify triggers for initiating ACP and the ‘optimal time window to initiate ACP’.^22^ Our research questions are (1) ‘What is the prevalence of triggers for initiating ACP, as operationalised for routine data, and is the presence of these triggers associated with death? and (2) ‘What is their position relative to a previously identified “optimal time window for ACP”?’

## Methods

### Study design and population

**Figure 1:**
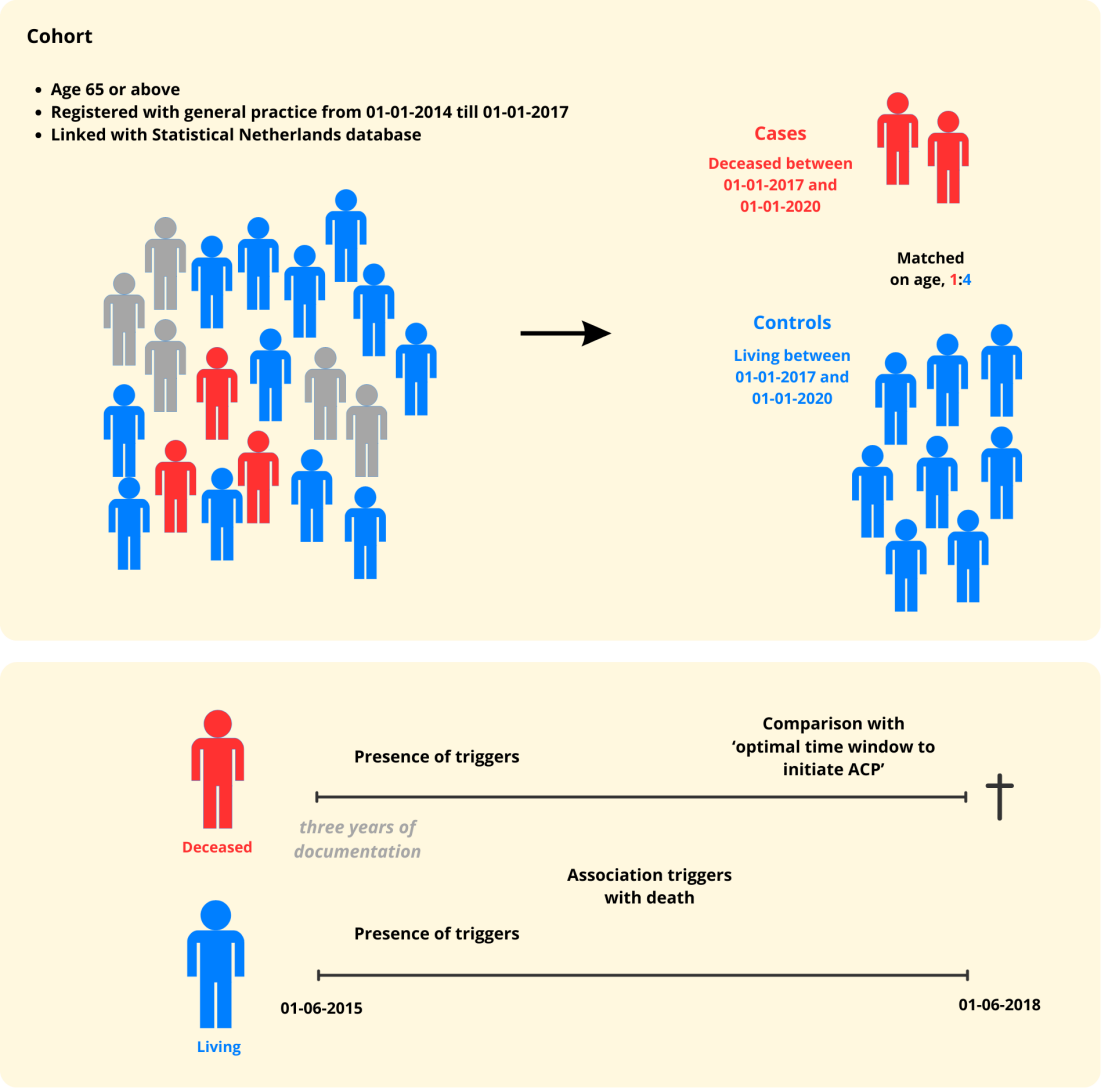
Study design and analysis. ACP, advance care planning.

This nested case-control study used Dutch routine primary healthcare data linked with national Statistics Netherlands data from the Extramural LUMC Academic Network (ELAN). ELAN is a regional integrative population-based data infrastructure encompassing medical, social and public health data from the South Holland province.^23^ Records from individuals were included in our study if they (1) were linked with the database of Statistics Netherlands (for birth- and death registration and referral information), (2) were registered with their practices from 1 Jan 2014 to 1 Jan 2017 (based on ELAN data) and (3) had an age of 65 or above as of 1 Jan 2017 (based on Statistics Netherlands data). We followed these individuals from 1 Jan 2017 to 1 Jan 2020 and categorised them into two groups: those who were deceased (both non-accidental and accidental deaths) by the end of the follow-up period (cases) and those who remained alive and continuously registered with their practice throughout the follow-up period (controls) (figure 1). Last, we matched the deceased individuals (cases) to living individuals (controls) by age in a 1:4 ratio using propensity score matching, applying nearest-neighbour matching with a 0.2 SD calliper. This method pairs each case with the nearest control within the specified calliper range (0.2 SD), ensuring matched individuals are similar in their propensity scores and thus sufficiently similar in age for our descriptive study. Despite a reduction of the standardised mean difference (SMD) from 1.079 pre-matching to 0.348 post matching, the desirable SMD of <0.1 was not achieved. However, we prioritised retaining the larger sample size. We considered age the most important factor likely to influence the overall prevalence of triggers (figure 1). We did not match on additional criteria for specific triggers to preserve the population’s similarity to a GP practice population. The study period was chosen to select data collected before the COVID-19 pandemic, as this might have affected the consultation behaviour of patients and the registration by GPs of data and thus the prevalence of ACP triggers. The age criterion was applied as in practice ACP is usually initiated with older individuals, and we expect a future algorithm to be implemented in these older individuals. We did not exclude accidental deaths, as we aimed for the study population to reflect the real-world general population. Regardless, given the low proportion of accidental deaths among the older population (<0.5% in 2017, 2018 and 2019), we do not expect their inclusion to have a material impact on findings.^24^

The ‘optimal time window’ had been identified in a previous study^22^ by presenting anonymised routine electronic health records of patients who died from cancer, organ failure or multimorbidity to participating general practitioners (three records per GP). Participants were asked to determine the optimal moment to initiate advance care planning in individual health records. We then expressed the results as the median number of days (with IQR) between the proposed time point and the patient’s death across all reviewed records (median optimal ACP timing 228 days before death, 25th–75th percentile 65–458 days before death).

### Data collection

We translated previously defined clinical indicators for initiating ACP^13^ into triggers in primary healthcare data and Statistics Netherlands data, excluding free-text data (online supplemental file 1). WT (involved in the previous study’s data analysis), PvP (GP and researcher) and MN (GP and researcher) translated the broad clinical indicators into more specific coded variables available in the primary healthcare data. This process integrated the researchers’ knowledge of the clinical indicators from previous study with the GPs’ practical experience of how such information is typically recorded in primary care. From primary healthcare data, we extracted consultation registrations according to the WHO International Classification of Primary Care (ICPC),^25^ laboratory test results, registered correspondence with paramedics and prescribed medication according to the Anatomical Therapeutic Chemical (ATC).^26^ From the Statistics Netherlands data, we extracted referral to the emergency department and hospital admission data. We extracted all data covering 3 years for everyone, 3 years preceding death for deceased individuals and 3 years preceding 1 June 2018 (midway the follow-up period) for individuals who were alive at the end of the follow-up period (figure 1). Data linkage was performed using record identification numbers. Data was selected, operationalised (online supplemental tables S1 and S2), linked and analysed within a protected remote access environment of Statistics Netherlands.

### Statistical analysis

We summarised categorical data as percentages. For continuous data, summary statistics of mean and SD are presented. We calculated the prevalence of triggers in records of deceased and living individuals as the percentage of individuals with at least one occurrence of each trigger during the 3-year follow-up. We excluded triggers with overall prevalence <1% from further analysis (online supplemental table S2). A univariable logistic regression model was estimated to study the association of a trigger’s presence with death; ORs along with 95% CIs are reported. Additionally, for triggers with an OR of 1.5 or higher,^27^ we described the timing of the triggers (days before death, median and 25th–75th percentile) in the records of deceased individuals relative to the ‘window of optimal ACP timing’ from our previous study.^22^ For all analyses, we used SPSS.

### Patient and public involvement

Neither patients nor public representatives were involved in the design, implementation or analysis of this study owing to constraints on funding and time.

## Results

**Figure 2:**
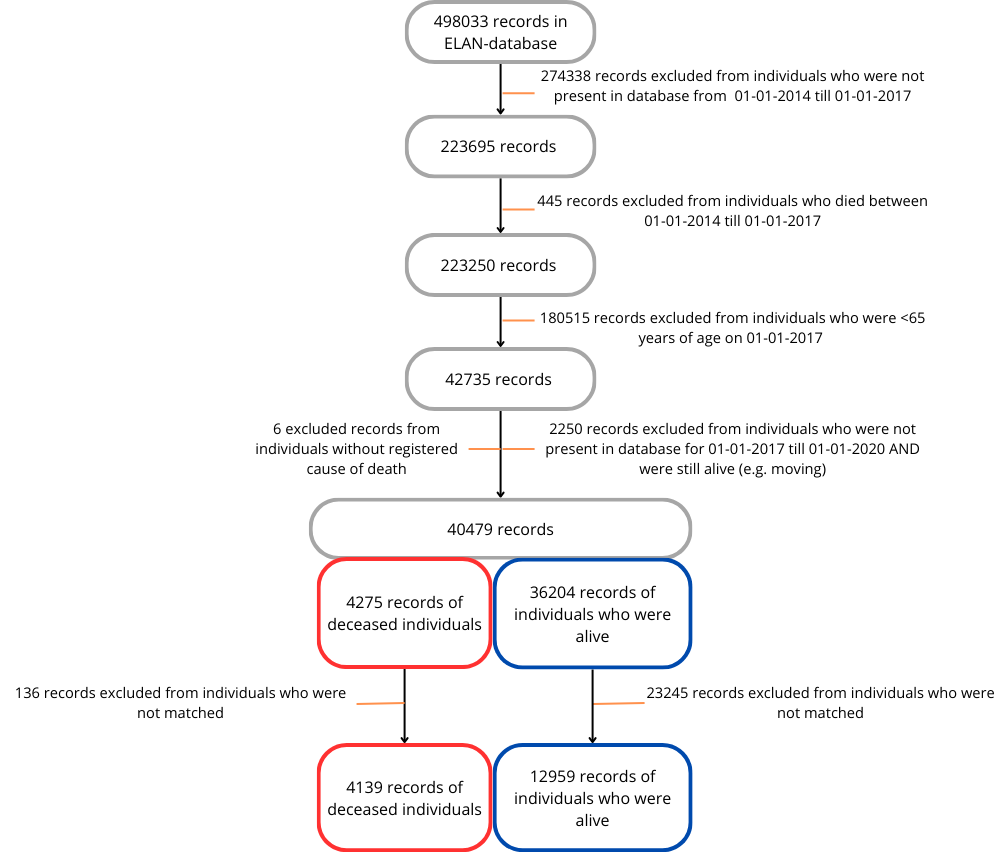
Selection of health records.

After applying our inclusion criteria, a total of 17098 records of individuals were used in the analysis, with 4139 records of deceased individuals and 12 959 records of individuals who were alive after the follow-up period (figure 2). Baseline characteristics of all individuals are presented in table 1.

**Table 1:** Characteristics of individuals from included records

	Deceased individuals* (n=4139)	Living individuals* (n=12 959)
Age in years‡, *mean (SD*)	81 (8)	79 (7)
Sex, *% (n*) Female	49.7% (2059)	57.7% (7479)
Age at death, *mean (SD*)	83 (8)	–
Causes of death,† *% (n*)	Malignant (34%, n=1399) Pulmonal or bronchial 7% (n=305) Colon 3% (n=106) Prostate 2% (n=102) Pancreatic 2% (n=99) Breast 2% (n=81) Non-malignant (66%, n=2740) Dementia, including Alzheimer’s disease 7% (n=288) COPD 5% (n=215) Heart failure 5% (n=206) Stroke 5% (n=193) Acute myocardial infarction 4% (n=172) Pneumonia 3% (n=124) Falls 3% (n=117) Non-specified cause of death 2% (n=102)	–

* This period covered 1 Jan 2017 until 1 Jan 2020.

† Based on ICD-10.

‡ Calculated at 1 Jan 2017.

COPD, chronic obstructive pulmonary disease.

### Prevalence and timing of triggers for ACP initiation

We show the triggers in the following sections by type of registered information in the EHR: first, registered consultations; second, medication prescriptions; and finally, other types of registered information like lab measurements and correspondence with paramedics.

### Registered consultations

**Figure 3:**
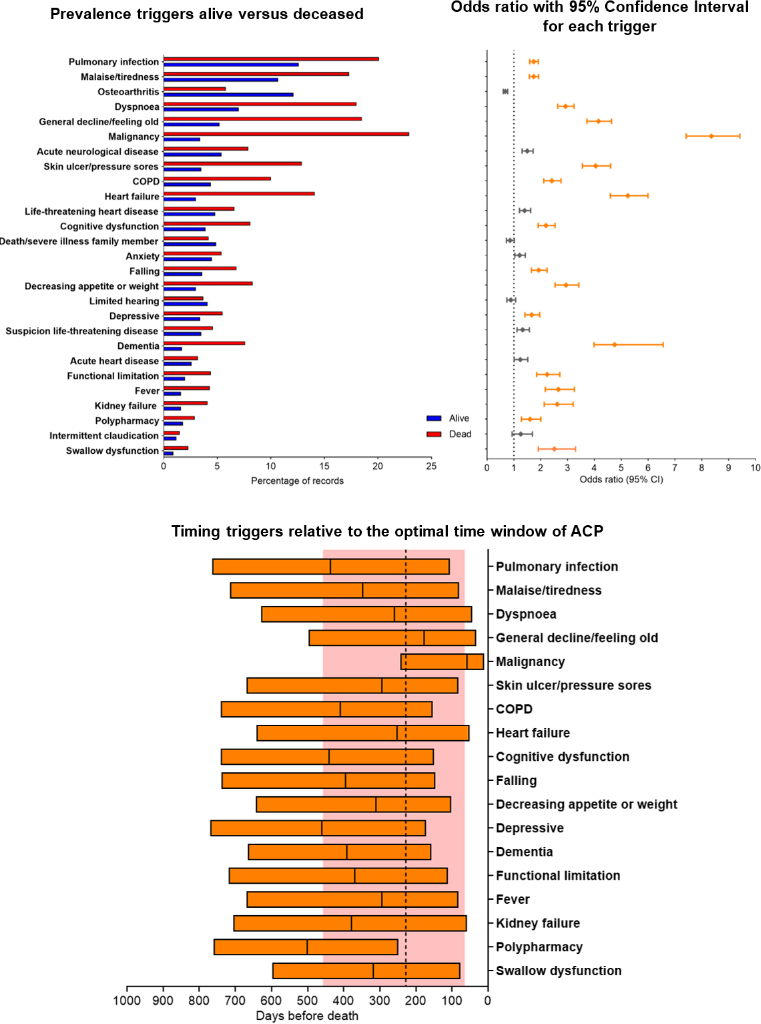
Top left panel: prevalence of each trigger in records from alive versus deceased individuals. Top right panel: estimated logistic regression for the outcome death; OR along with 95% CI for triggers; ORs <1.5 and >1.5 are coloured in grey and orange, respectively. Bottom middle panel: timing (median and IQR) of a selection of triggers (OR>1.5) versus the optimal time window of ACP (red area and vertical dotted line). This optimal time window of ACP had been identified by GPs in a previous study in records from patients who died of cancer, organ failure or multimorbidity. ACP, advance care planning; COPD, chronic obstructive pulmonary disease; GPs, general practitioners.

The five most prevalent registered consultations were consultations for pulmonary infection, malaise/tiredness, osteoarthritis, dyspnoea and general decline/feeling old (figure 3, online supplemental file 1). Almost all registered consultations were positively associated with death, except consultations for osteoarthritis, death/severe illness of a partner/family member, limited hearing and intermittent claudication. The five registered consultations with the highest OR were consultations concerning malignancy (OR 8.35, 95% CI 7.42 to 9.41), heart failure (OR 5.25, 95% CI 4.59 to 5.99), dementia (OR 4.75, 95% CI 3.99 to 6.56), general decline/feeling old (OR 4.15, 95% CI 3.72 to 4.64) and skin ulcers/pressure sores (OR 4.04, 95% CI 3.55 to 4.61). The five registered consultations with the median timing closest to the median of the optimal time window for ACP were consultations regarding dyspnoea, general decline/feeling old, heart failure, skin ulcers/pressure sores and fever. Registered consultations regarding dementia, pulmonary infection, chronic obstructive pulmonary disease (COPD), cognitive dysfunction, polypharmacy and depressive symptoms were timed relatively earlier compared with the optimal ACP timing.

### Medication registration

**Figure 4:**
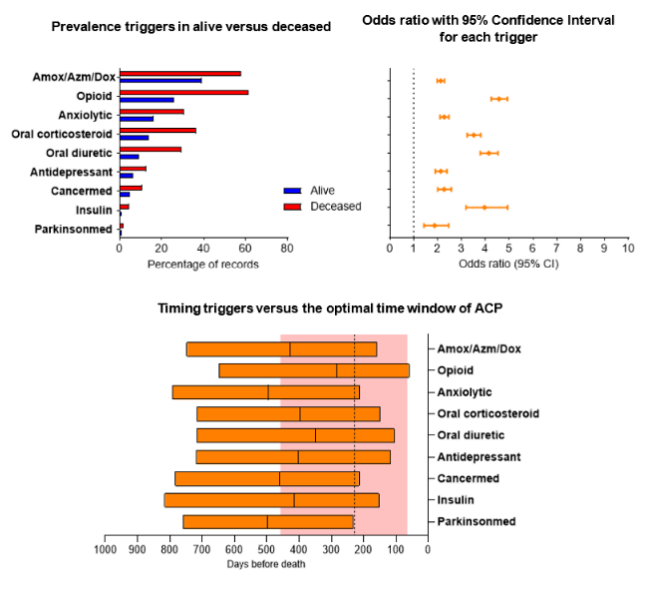
Top left panel: prevalence of each trigger in records from alive vs deceased individuals. Top right panel: estimated logistic regression for the outcome death; OR along with 95% CI for triggers; ORs <1.5 and >1.5 are coloured in grey and orange respectively. Bottom middle panel: timing (median and IQR) of a selection of triggers (OR>1.5) versus the optimal time window of ACP (red area and vertical dotted line). This optimal time window of ACP is in a previous study identified by GPs in records from patients died of cancer, organ failure or multimorbidity. ACP, advance care planning; Amox/Azm/Dox, amoxicillin, azithromycin and doxycycline; GPs, general practitioners.

The two most prevalent medication prescriptions were for the antibiotics amoxicillin, azithromycin or doxycycline, and for opioids (figure 4). All medication prescriptions were positively associated with death. Prescriptions of opioids had the highest odds ratio (OR 4.58, 95% CI 4.25 to 4.93) and its median timing was closest to the median of the optimal time window for ACP. Further, prescriptions of oral corticosteroids (OR 3.51, 95% CI 3.24 to 3.81), oral diuretics (OR 4.15, 95% CI 3.79 to 4.53) and insulin (OR 3.97, 95% CI 3.19 to 4.94) had a high OR; however, these are relatively early timed compared with the optimal ACP timing.

### ‘Other’ registration

**Figure 5:**
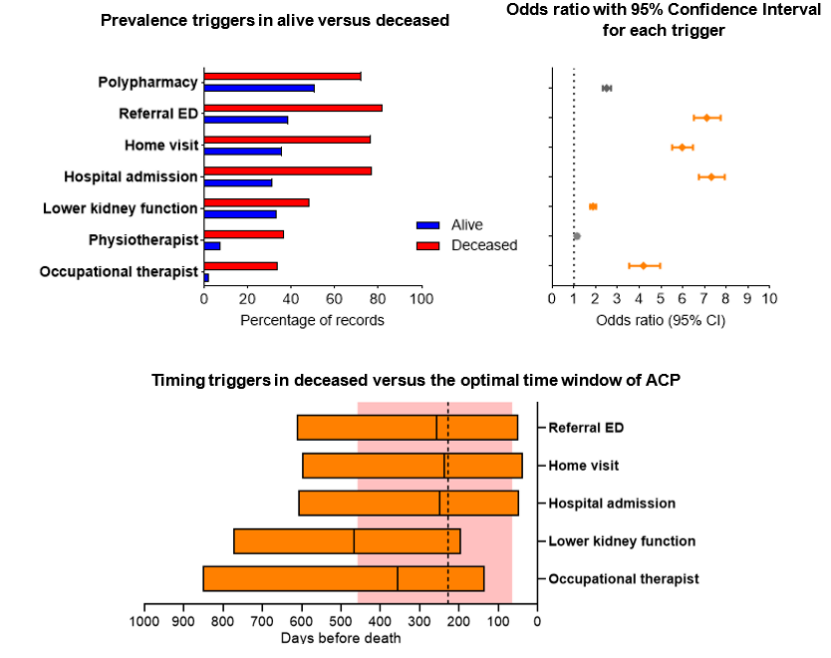
Top left panel: prevalence of each trigger in records from alive vs deceased individuals. Top right panel: estimated logistic regression for the outcome death; OR along with 95% CI for triggers; ORs <1.5 and >1.5 are coloured in grey and orange, respectively. Bottom middle panel: timing (median and IQR) of a selection of triggers (OR>1.5) versus the optimal time window of ACP (red area and vertical dotted line). This optimal time window of ACP is in a previous study identified by GPs in records from patients died of cancer, organ failure or multimorbidity. ACP, advance care planning; Referral ED, referral to emergency department.

Almost all other triggers we studied were prevalent in the included records and are associated with death. An emergency department referral (OR 7.11, 95% CI 6.52 to 7.75), a registered home visit (OR 5.97, 95% CI 5.51 to 6.47) and a hospital admission (OR 7.32, 95% CI 6.75 to 7.94) had the highest OR and a median timing closest to the median of the optimal time window for ACP. The lower kidney function and a correspondence with an occupational therapist appeared earlier in the records (figure 5).

### Total number of triggers in included records over follow-up period

**Figure 6:**
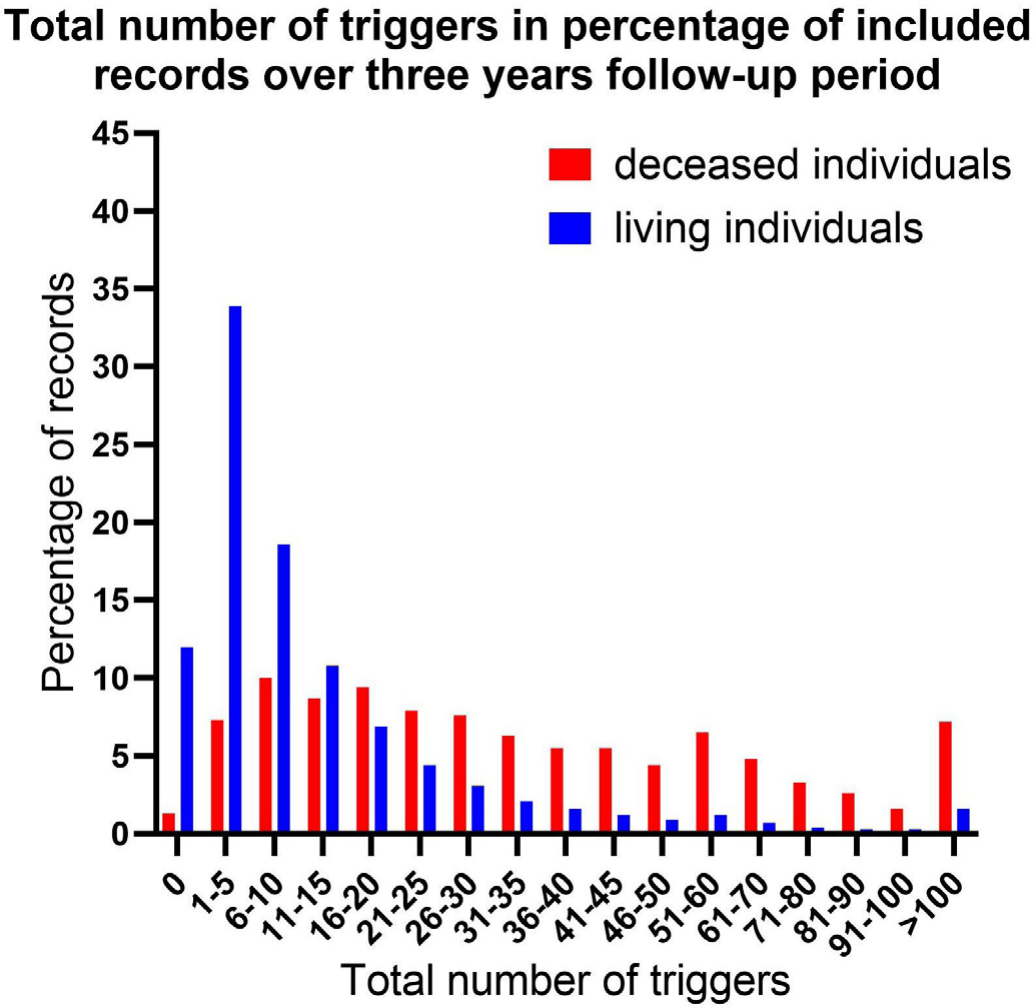
Total number of triggers in percentage of records of deceased versus living individuals. Analysis performed on all triggers with an OR of >1.5.

Figure 6 shows the distribution of records by the total number of ACP triggers in deceased individuals versus those still alive. For records with more than a total of 15 triggers, the proportion is higher in the records of deceased individuals compared with the records of individuals who were still alive.

## Discussion

### Principal findings

We found that triggers for initiating ACP can be identified in EHRs of older individuals in general practice. Most triggers were associated with the outcome death. Overall, the timing of the triggers, as operationalised for routine data, aligns well with the previously defined ‘window of optimal timing of ACP’. Notable triggers, strongly associated with death (ie, high OR) and a median timing close to this window, are registered consultations regarding dyspnoea, general decline/feeling old, heart failure, skin ulcers/pressure sores and fever, registered opioid prescriptions, registered home visits, referrals to the emergency department and hospital admissions. These triggers could prompt immediate initiation of ACP as they occur. Further, we found that many triggers had a median timing positioned relatively early relative to the window of optimal ACP timing, such as consultations regarding pulmonary infection, COPD, cognitive dysfunction, depressive symptoms, oral diuretics and oral corticosteroid prescriptions and measurement of kidney function decline. These could require action when multiple numbers of triggers have occurred.

### Strengths and weaknesses

Our study has several strengths. First, we focused on triggers for initiating ACP rather than for imminent death, resulting in the identification of a broader scope of potentially valuable triggers, adding to prior literature. Second, the analysis encompassed an entire general practice population rather than a population limited to a specific disease group. By using real-life data, we ensured that our findings are applicable in clinical practice, where identification tools ultimately need to be implemented. Also, our time frame of identifying triggers up to 3 years before death captured a variety of relevant triggers.

However, some limitations should be acknowledged. ACP is not defined as inclusive of end-of-life communication^7 28^ but we pragmatically choose end of life as a reference in our analyses. Further, our study relies on routinely recorded primary care data and coded data only, which may result in underreporting or missing entries. We mitigated part of this issue by incorporating national data of Statistics Netherlands. Additionally, we acknowledge that the manner and extent of registration may have evolved between our study period (2017–2020) and today, which could potentially result in differences in outcomes. Nonetheless, the key triggers we identified remain relevant to current practice. Last, our cohort of urban and rural areas in South Holland with a mean age of around 80 may not be fully representative of the countries’ older primary care population. Matching with the deceased individuals resulted in a relatively old study population, and the prevalence of triggers found in this sample may not be generalisable to other populations of patients above age 65. However, we expect that triggers will be less present in a younger control group. Moreover, we have selected a group of patients who remained in a single GP practice for the whole study period to ensure sufficient longitudinal follow-up.

### Comparison with existing literature

Our findings align with existing research, highlighting both the potential and limitations of using routinely collected healthcare data for identifying end of life, palliative care needs or declining health status.^20 21 29 30^ Gebresillassie *et al* emphasise the greater potential of generic predictors, such as functional decline and cognitive impairment, over disease-specific ones, as reflected in our results.^20^

The heterogeneity of disease and ageing trajectories, as reflected in the diversity of timing of the triggers in our study and also noted by Gebresillassie *et al*,^20^ complicates creating universally applicable predictive models, especially for such a complicated subject as ACP. Furthermore, the essential role of clinical judgement of GPs remains difficult to capture in future predictive models and algorithms.

Mason *et al* provide a practical example of integrating an automated computer search into clinical practice to identify patients with palliative care needs and thus people who are candidates for ACP.^21^ Their search algorithm combined with clinical judgement was effective, especially in identifying patients with multimorbidity or frailty─a population where ACP is underutilised─as that aligns with the complexity the best as possible.

### Implications for practice and future research

This study could lay the foundation for further development of automated tools in the EHR to guide ACP initiation in clinical practice. GPs can use the triggers identified in the EHR in an automated computer search to prioritise patients for ACP. They may prioritise ACP in patients with multiple triggers present in their EHR, particularly those more present in deceased patients and with closest proximity to the optimal ACP timing window including registered consultations regarding dyspnoea, general decline/feeling old, heart failure, skin ulcers/pressure sores, fever, opioid prescriptions, home visits, emergency department referrals and hospital admissions. Immediate ACP initiation may be warranted on detecting these triggers. Other triggers we identified may be combined to serve as a cumulative numerical indicator, warranting consideration to initiate ACP when a total number of >15 (repetitive) triggers are present. A critical next step involves implementing automated identification tools based on triggers for ACP to evaluate its integration into clinical practice and its clinical impact.

### Conclusions

Clinical triggers for initiating ACP in general practice can be recognised within routinely collected electronic health records, and they align well with the ‘window of opportunity’ to initiate ACP. Some triggers may be more suitable for prompting immediate initiation of ACP when they occur, while others may require action only when multiple triggers have occurred. Furthermore, the identified triggers in the electronic health records could be used in the further development of automated tools to guide identifying individuals who could benefit from initiation of ACP.

## Supplementary material

10.1136/bmjopen-2025-104742 Supplementary file 1

## Data Availability

No data are available.
