# Supplementary material for: Identifying triggers for optimal timing of advance care planning in electronic primary health care records: a nested case-control study
Source: BMJ Open. 2025 Nov 29;15(11):e104742. doi: 10.1136/bmjopen-2025-104742 (PMC12666204; doi:10.1136/bmjopen-2025-104742)
Supplement: Supplementary file 1 [file bmjopen-15-11-s001.docx]

**Supplemental file**

**Table S1.** The explored triggers, derived from clinical indicator to initiate ACP.

| Trigger | ICPC’s or ATC code(s) + Label | Data adjustments* | Derived from clinical indicator previous study (1) | Prevalence in health records**, in % | Association with the outcome of death, *odds ratio (95% CI)* |
| --- | --- | --- | --- | --- | --- |
| *Registered consultations (home visits, consultations or consultations via telephone)* | | | |  |  |
| Pulmonary infection | - R78 Acute bronchitis  - R81 Pneumonia | Multiple consultations a day for a single ICPC counted as one consultation | Acute symptoms | 14.4 | 1.74 (1.59-1.91) |
| Malaise/tiredness | A04 Malaise and fatigue | Multiple consultations a day for a single ICPC counted as one consultation | General decline | 12.3 | 1.74 (1.58-1.92) |
| Osteoarthritis | - L89 Hip osteoarthritis  - L90 Knee osteoarthritis  - L91 Other osteoarthritis/ related conditions  - L88 Rheumatism | Multiple consultations a day for a single ICPC counted as one consultation | Functional decline | 11.2 | 0.68 (0.60-0.77) |
| Dyspnoea | R02 Dyspnoea/breathlessness attributed to the respiratory system | Multiple consultations a day for a single ICPC counted as one consultation | Acute symptoms | 9.7 | 2.92 (2.63-3.24) |
| General decline/feeling old | - A05 General decline  - P05 Feeling old | Multiple consultations a day for a single ICPC counted as one consultation | General decline | 8.4 | 4.15 (3.72-4.64) |
| Malignancy | - A79 Malignancy with unknown primary site  - B72 Hodgkin’s’ disease (B72.01+ B72.02)  - B73 Leukaemia  - B74 Other malignancies blood/lymphatic system  - D74 Malignancy stomach  - D75 “ colon/rectum  - D76 “ pancreas  - D77 Other/unspecified malignancies digestive system  - F74.01 “ eye/adnexa  - H75.01 “ ear  - K72.01 “ cardio-vascular system  - L71.01 “ musculo-skeletal system  - N74 “ nervous system  - R84 “ bronchus/lung  - R85 Other malignancies respiratory system  - S77.02 Squamous cell carcinoma skin  - S77.03 Malignant melanoma  - S77.04 Kaposi's sarcoma  - T71 “ thyroid  - U75 “ kidney  - U76 “ bladder  - U77 Other malignancies urinary system  - W72 “ associated with pregnancy  - X75 “ cervix uteri  - X76 + X76.01 “ female breast  - X77 Other malignancies female genital organs (X77.01 and X77.02)  - Y77 “ prostate  - Y78 + .01 Other malignancies male genital organs/breasts | Multiple consultations a day for a single ICPC counted as one consultation | Diagnosis acute or chronic life- threatening illness | 8.1 | 8.35 (7.42-9.41) |
| Acute neurological disease | - K89 TIA (Transient Ischaemic Attack)  - K90 Stroke  - K90.01 Subarachnoid haemorrhage  - K90.02 Intracerebral haemorrhage  - K90.03 Cerebral infarction | Multiple consultations a day for a single ICPC counted as one consultation | After experiencing an acute illness or potentially life-threatening moment | 6.0 | 1.50 (1.31-1.72) |
| Skin ulcer/pressure sore | S97 Chronic leg ulcer/pressure sore | Multiple consultations a day for a single ICPC counted as one consultation | Functional decline | 5.8 | 4.04 (3.55-4.61) |
| COPD | R95 Emphysema/COPD | Multiple consultations a day for a single ICPC counted as one consultation | Diagnosis acute or chronic life- threatening illness | 5.8 | 2.41 (2.12-2.75) |
| Heart failure | - K77 Heart failure  - K77.01 Acute heart failure  - K77.02 Chronic heart failure | Multiple consultations a day for a single ICPC counted as one consultation | Diagnosis acute or chronic life- threatening illness | 5.7 | 5.25 (4.59-5.99) |
| Life-threatening heart disease | - K99.01 Aortic aneurysm  - K78 Atrial fibrillation/flutter | Multiple consultations a day for a single ICPC counted as one consultation | Diagnosis acute or chronic life- threatening illness | 5.2 | 1.40 (1.21-1.62) |
| Cognitive dysfunction | P20 memory/concentration /orientation disturbances | Multiple consultations a day for a single ICPC counted as one consultation | Cognitive dysfunction | 4.9 | 2.20 (1.90-2.53) |
| Death/severe illness family member | - Z15 loss/death of partner  - Z14 problem with partner’s illness  - Z19 loss/death of child  - Z23 loss/death of parents/family | Multiple consultations a day for a single ICPC counted as one consultation | Death/severe illness family member | 4.7 | 0.86 (0.72-1.02) |
| Anxiety | - P01 anxious/nervous/tense  - P74 anxiety disorder | Multiple consultations a day for a single ICPC counted as one consultation | Emotional expressions from the patient/family | 4.7 | 1.22 (1.04-1.42) |
| Falling | A06 fainting/syncope | Multiple consultations a day for a single ICPC counted as one consultation | Red flag symptoms | 4.4 | 1.92 (1.65-2.24) |
| Decreasing appetite or weight | - T03 reduced appetite  - T08 weight loss | Multiple consultations a day for a single ICPC counted as one consultation | Red flag symptoms | 4.3 | 2.94 (2.53-3.42) |
| Limited hearing | - H86 Deafness/hearing impairment  - H84 Presbycusis | Multiple consultations a day for a single ICPC counted as one consultation | Functional decline | 4.0 | 0.74 (0.89-1.07) |
| Depressive | - P03 feeling down/depressed  - P76 depressive disorder | Multiple consultations a day for a single ICPC counted as one consultation | Emotional expressions from the patient/family | 3.9 | 1.66 (1.41-1.96) |
| Suspicion life-threatening disease | - R24 Haemoptysis  - X19 Lump/swelling in female breast  - X12 Postmenopausal bleeding  - X86 Abnormal cervical smear  - D18 Change in defecation pattern | Multiple consultations a day for a single ICPC counted as one consultation | Suspicion life-threatening disease | 3.7 | 1.33 (1.12-1.58) |
| Dementia | - P70 Senile dementia  - P70.01 Alzheimer’s disease  - P70.02 Multi-infarct dementia | Multiple consultations a day for a single ICPC counted as one consultation | Diagnosis acute or chronic life- threatening illness | 3.1 | 4.75 (3.99-6.56) |
| Acute heart disease | - K99.01 Aortic aneurysm  - K75 Acute myocardial infarction  - K76.02 Previous myocardial infarction | Multiple consultations a day for a single ICPC counted as one consultation | After experiencing an acute illness or potentially life-threatening moment | 2.7 | 1.24 (1.01-1.52) |
| Functional limitation | - A28 Functional limitation/disability  - B28 “ Blood/lymphatic system  - D28 “ Digestive system  - F28 “ Eye/adnexa  - H28 “ Ear  - K28 “ Cardiovascular system  - L28 “ Musculoskeletal system  - N28 “ Nervous system  - R28 “ Respiratory system  - U28 “ Urinary system  - Z28 “ Social | Multiple consultations a day for a single ICPC counted as one consultation | Functional decline | 2.6 | 2.24 (1.85-2.71) |
| Fever | A03 Fever | Multiple consultations a day for a single ICPC counted as one consultation | Acute symptoms | 2.3 | 2.66 (2.17-3.26) |
| Kidney failure | U99.01 Renal dysfunction/insufficiency | Multiple consultations a day for a single ICPC counted as one consultation | Diagnosis acute or chronic life- threatening illness | 2.2 | 2.61 (2.13-3.21) |
| Polypharmacy | A49.02 polypharmacy | Multiple consultations a day for a single ICPC counted as one consultation | Polypharmacy | 2.1 | 1.60 (1.28-2.00) |
| Intermittent claudication | K92.01 Intermittent claudication | Multiple consultations a day for a single ICPC counted as one consultation | Acute symptoms | 1.3 | 1.26 (0.93-1.69) |
| Swallow dysfunction | - D21 Swallowing problems  - D84.05 Oesophageal obstruction | Multiple consultations a day for a single ICPC counted as one consultation | Red flag symptoms | 1.2 | 2.51 (1.91-3.29) |
| *Medication prescription* | | | | | |
| Amox/Azm/Dox | - J01CR02 Amoxicillin and beta-lactamase inhibitor  - J01CA04 Amoxicillin  - J01FA10 Azithromycin  - J01AA02 Doxycycline | Prescription of any of the four medications and no other such prescriptions in the past 14 days | Acute symptoms | 43.6 | 2.13 (1.99-2.29) |
| Opioids | N02A Opioids | Prescription of any N02A-medication, multiple prescriptions a day for any N02A-medication counted as one prescription | Red flags symptoms | 34.5 | 4.58 (4.25-4.93) |
| Anxiolytic | N05B Anxiolytics | Prescription of any N05B-medication, multiple prescriptions a day for any N05B-medication counted as one prescription | Emotional expressions from the patient/family | 19.7 | 2.28 (2.11-2.48) |
| Oral corticosteroids | - H02AB07 Prednisone  - H02AB06 Prednisolone  - H02AB02 Dexamethasone | Prescription of any of the three medications, multiple prescriptions a day for any of these medications counted as one prescription | Exacerbation organ failure | 19.5 | 3.51 (3.24-3.81) |
| Oral diuretics | - C03CA01 Furosemide  - C03CA02 Bumetanide | Prescription of any of the two medications and no other such prescriptions in the past 6 months | Exacerbation organ failure | 14.0 | 4.15 (3.79-4.53) |
| Antidepressant | N06A antidepressants | Prescription of any of the N06A-medication and no other such prescriptions in the past 6 months | Emotional expressions from the patient/family | 7.9 | 2.13 (1.90-2.40) |
| Cancer medication | - L01 Antineoplastic agents  - L02 Endocrine therapy used specifically in neoplasms | Prescription of any of the L01- or L02-medication and no other such prescriptions in the past year | Diagnosis acute or chronic life- threatening illness | 6.5 | 2.27 (2.00-2.58) |
| Insulin | A10A Insulins and analogues | Prescription of any of the A10A-medication and no other such prescriptions in the past year | Deterioration of chronic disease | 2.0 | 3.97 (3.19-4.94) |
| Medication Parkinson’s disease | N04 Anti-Parkinson drugs | Prescription of any of the N04-medication and no other such prescriptions in the past year | Diagnosis acute or chronic life- threatening illness | 1.3 | 1.87 (1.43-2.46) |
| *‘Other’ registrations* | | | | | |
| Polypharmacy | - | ≥ 5 different drugs, prescribed; at least≥ 3 in past year and ≥1 past 6 months, assessed at last day of the year (31^st^ of December) | Polypharmacy | 56.0 | 2.51 (2.32-2.70) |
| Referral emergency department (ED) | - | Consultation with the emergency department, multiple consultations a day counted as one | After experiencing an acute illness or potentially life-threatening moment | 49.2 | 7.11 (6.52-7.75) |
| Registered home visit | - | A registered consultation with consultation type ‘home visit’, multiple home visits a day counted as one | Functional decline/ general decline | 45.5 | 5.97 (5.51-6.47) |
| Hospital admission | - | First day of a series of consecutive hospitalization days | After experiencing an acute illness or potentially life-threatening moment | 42.3 | 7.32 (6.75-7.94) |
| Measurement lower kidney function | - | MDRD or CKD-EPI <60 (and >1.0), multiple measurements a day counted as one (CKD-EPI is leading) | Diagnosis acute or chronic life-threatening illness | 37.1 | 1.88 (1.75-2.02) |
| Referral physiotherapy | - | Correspondence*** with physiotherapist, multiple correspondences a day counted as one | Functional decline | 34.3 | 1.14 (1.06-1.22) |
| Referral occupational therapist | - | Correspondence with occupational therapist, multiple correspondences a day counted as one | Functional decline | 3.3 | 4.19 (3.54-4.97) |

* raw coded information was adjusted to make practical coded information for analysis

** the percentage of individuals with at least one occurrence of each trigger

*** a written digital communication such as letters and messages

**Table S2.** Triggers excluded from further analysis due to a prevalence of <1% in records

| Trigger | ICPC’s or ATC code(s) + Label | Data adjustments* | Derived from clinical indicator previous study (1) |
| --- | --- | --- | --- |
| *Registered consultations* | | | |
| Chronic progressive neurological disease | - N86 Multiple sclerosis  - N87 + .01 Parkinson’s disease  - N99.01 ALS (Amyotrophic Lateral Sclerosis) | Multiple consultations a day for a single ICPC counted as one consultation | Diagnosis acute or chronic life- threatening illness |
| Liver failure | - D97.04 Cirrhosis | Multiple consultations a day for a single ICPC counted as one consultation | Diagnosis acute or chronic life- threatening illness |
| Palliative care | - A69.01 Palliative care | Multiple consultations a day for a single ICPC counted as one consultation | No curative treatment options remaining |
| Dependence on care | - Z10.02 Waiting for a place in a care/nursing home  - Z29.03 Dependence on others | Multiple consultations a day for a single ICPC counted as one consultation | Functional decline |
| Blindness | - F94 Blindness (any degree/form) | Multiple consultations a day for a single ICPC counted as one consultation | Functional decline |
| Acute and severe gastrointestinal symptoms | - D99.01 Ileus  - K92.01 Pancreatitis | Multiple consultations a day for a single ICPC counted as one consultation | Acute symptoms |
| Acute vascular disease | - K93 Pulmonary embolism  - K94.01 DVT (Deep Vein Thrombosis) | Multiple consultations a day for a single ICPC counted as one consultation | Acute symptoms |
| Hip fracture | L75 Femoral fracture | Multiple consultations a day for a single ICPC counted as one consultation | Acute symptoms |
| Gastrointestinal blood loss | - D14 haematemesis  - D15 melaena | Multiple consultations a day for a single ICPC counted as one consultation | Acute symptoms |
| Deterioration of chronic disease | D29.01 ascites | Multiple consultations a day for a single ICPC counted as one consultation | Deterioration of chronic disease |
| Fear of illness/death | - A25 fear of death  - A26 fear of cancer  - B26 “ blood/lymphatic system  - D26 “ digestive organs  - L26 “ musculoskeletal system  - N26 “ nervous system  - R26 “ respiratory system  - S26 “ skin/subcutis  - T26 “ endocrine glands  - U26 “ urinary system  - X26 “ female genital organs  - Y26 “ male genital organs | Multiple consultations a day for a single ICPC counted as one consultation | Emotional expressions from the patient/family |
| *Medication prescriptions* | | | |
| Diuretics injections | - C03CA01 Furosemide (parenteral)  - C03CA02 Bumetanide (parenteral) | Prescription of any of these two medication, multiple prescriptions a day for any of these medications counted as one prescription | Exacerbation organ failure |
| Dementia medication | N06D anti-dementia drugs | Prescription of any of the N06D-medication and no other such prescriptions in the past year | Diagnosis acute or chronic life- threatening illness |
| MS medication | - L04AA23/-27/-31/-34/-36/-38/-40/-42/-50/-52 Selective immunosuppressants  - L03AX13 Glatiramer acetate (immunostimulants)  - L03AB07/-08/-13 Interferons | Prescription of any of the MS-medications and no other such prescriptions in the past year | Diagnosis acute or chronic life- threatening illness |
| ALS medication | N07XX02 Riluzole | No other riluzole prescriptions in the past year | Diagnosis acute or chronic life- threatening illness |
| *‘Other’ registrations* | | | |
| Referral mental health |  | Correspondence** with physiotherapist, multiple correspondences a day counted as one | Emotional expressions from the patient/family |
| Lowered oxygen saturation measurement |  | O2 saturation of <94 | Exacerbation organ failure / Acute symptoms |
| Fever measurement |  | Temperature of ≥38 and <42 | Acute symptoms |

* raw coded information was adjusted to make practical coded information for analysis

** written digital communication such as letters and messages

1. Tros W, van der Steen JT, Liefers J, Akkermans R, Schers H, Numans ME, et al. General practitioners' evaluations of optimal timing to initiate advance care planning for patients with cancer, organ failure, or multimorbidity: A health records survey study. Palliat Med. 2021:2692163211068692.
