# Supplementary material for: Identifying triggers for optimal timing of advance care planning in electronic primary health care records: a nested case-control study
Source: BMJ Open. 2025 Nov 29;15(11):e104742. doi: 10.1136/bmjopen-2025-104742 (PMC12666204; doi:10.1136/bmjopen-2025-104742)
Supplement: Supplementary file 2 [file bmjopen-2025-104742.reviewer_comments.pdf]

PEER REVIEW HISTORY

BMJ Open publishes all reviews undertaken for accepted manuscripts. Reviewers are asked to complete a checklist review form and are provided with free text boxes to elaborate on their assessment. These free text comments are reproduced below.

ARTICLE DETAILS

Title (Provisional)

Identifying triggers for optimal timing of advance care planning in electronic primary health care records: a nested case-control study

Authors

Tros, Willemijn; van der Steen, Jenny; Numans, Mattijs E.; Fiocco, Marta; van Peet, Petra G.

VERSION 1 - REVIEW

**Reviewer**	**1**
**Name**	**Aliarzadeh, Babak**
**Affiliation**	**University of Toronto, Department of Family and Community Medicine**
**Date**	**04-Jul-2025**
**COI**	**None**

I have no additional comments

**Reviewer**	**2**
**Name**	**Lee, Yew Kong**
**Affiliation**	**Universiti of Malaya, Department of Primary Care Medicine**
**Date**	**29-Jul-2025**
**COI**	**None**

This study builds on authors' previous work to establish a series of triggers and an optimum window for ACP discussion, by comparing the prevalence and effect of the triggers and how early the triggers appeared in the medical records with the optimal window. It utilises a lot of real world data to further add to the model previously developed, however I have some questions as mentioned below.

- Although reference is made to the previous optimal window study, it might be helpful to describe what this means in terms of median days and window in Page 7 line 10 so that

readers are familiar with the concept and its units. It is also a bit strange that given the variety of triggers and expected lifespan variation in the previous study, that a single window and mean could be calculated as the optimum- I would have expected that GPs in the previous study would have been more careful in saying there is no one size fits all for ACP?

- For matching case-controls, is age alone enough for matching? Some justification should be given for using this as the matching criterion.

- From Table 1, it looks as if the case and control groups may have some baseline differences (ie the Living individuals appear to be younger in mean age, and more female). I wonder if these need to be controlled for in the regression. It might not change the outcomes, as the list of triggers make sense as being linked to higher mortality, but the effect may not be as accurate if the baseline confounders are not controlled for.

- It is unclear how the list of triggers that were derived from the previous study as the list given in this study are very detailed and disease specific, whereas the reference does not have such a detailed list. I was curious about this, as some triggers were supposed to be associated with death (since they were included) but had an opposite outcome (e.g. osteoporosis and physiotherapy), so I was wondering how these outcomes were deemed to be ACP triggers, but could not find the details for this in this paper or the reference. Perhaps some explanation on how the list of triggers was discussed and derived would help.

- As the window matching is based on a three year follow-up period (3 years before June 2018), some justification for this 3 year window should be given. It is quite striking that the median days fall largely within the optimal window for the deceased cases in the majority of triggers and I question whether this might be due to the 3 year window- perhaps if the records were analysed further back, the median days might go further back in terms of the trigger dates- for example medications might have been prescribed much earlier than the 3 years of the follow-up period. Some clarity on reasons for the 3 year window and how this might affect the median date of the trigger should be given.

- For Table 1, how was Age in years calculated? At date of first and last record, or date of death? How does this fit into the 3 year window period for example.

**Reviewer**	**3**
**Name**	**Kelson, Zoe**
**Affiliation**	**University of Exeter, Mathematics**
**Date**	**13-Aug-2025**
**COI**	**None**

This nested case-control study within a large dynamic population cohort dataset aims to explore whether routine electronic healthcare records can be used to identify triggers for initiating advance care planning (ACP) and the optimal time window to initiate ACP.

Reviewer comments:

"Triggers most strongly associated with death (i.e. highest odds ratios) were consultations concerning malignancy, heart failure, dementia, general decline/feeling old and skin ulcers/pressure sores; opioid prescriptions; emergency department referrals, registered home visits and hospital admissions. Those closest to the median of the optimal time window for ACP were consultations regarding dyspnea, general decline/feeling old, heart failure, skin ulcers/pressure sores and fever; opioid prescriptions; emergency department referrals, registered home visits and hospital admissions."

Can statistical outcomes please be reported alongside study findings in the Abstract?

"Primary care population-based, anonymized data extracted from GP centers in the South-Holland province, The Netherlands...

We selected records of individuals aged ≥ 65 registered with their general practice from 01-01-2014 till 01-01-2017"

and

"We included 17098 included records, 4139 from deceased individuals (mean age 81) and 12.959 from living individuals (mean age 79)."

and

"This nested case-control study utilized Dutch routine primary health care data linked with national Statistics Netherlands data from the Extramural LUMC Academic Network (ELAN). "

Can the authors please further comment on whether the remaining analysed cohort can be considered to be demographically and characteristically representative for generalisation of the study outcomes?

"Cases were individuals who died between 01-01-2017 and 01-01-2020. Controls were individuals who remained alive"

Can the authors please clarify how accidental (e.g. non-clinical, such as a car accident) deaths are accounted for in the analyses?

"Cases were matched by age to controls in a 1:4 ratio" [Abstract]

and

"we matched the deceased individuals (cases) to living individuals (controls) by age at a 1:4 ratio to minimize age-related bias (figure 1)"

Can the matching algorithm or process please be specified in more detail?

Can the authors please consider also matching on other factors, such as sex?

"The study period was chosen to select data collected before the COVID-19 pandemic, as this might have affected the consultation behavior of patients and the registration by GPs of data and thus the prevalence of ACP triggers."

Can the authors please comment on whether they consider study outcomes to be applicable today (i.e. post COVID-19 pandemic)?

"From primary healthcare data, we extracted consultation registrations according to the WHO International Classification of Primary Care (ICPC)(24), laboratory test results, registered correspondence with paramedics, and prescribed medication according to the Anatomical Therapeutic Chemical (ATC) (25). From the Statistics Netherlands data, we extracted referral to the emergency department- and hospital admission data. We extracted all data covering three years for everyone; three years preceding death for deceased individuals, and three years preceding 1st of June 2018 (midway the follow-up period) for individuals who were alive at the end of the follow-up period (figure 1). Data linkage was performed using record identification numbers. Data was selected, operationalized (Supplemental file, table S1 and table S2), linked, and analyzed within a protected remote access environment of Statistics Netherlands"

Can the authors please confirm if there was any missing data?

"A univariable logistic regression model was estimated to study the association of a triggers' presence with death; odds ratios along with 95% confidence are reported. Additionally, for triggers with an odds ratio of 1.5 or higher (26), we described the timing of the triggers (days before death, median and 25th-75th percentile) in the records of deceased individuals relative to the 'window of optimal ACP timing' from our previous study"

Can the authors please also conduct multivariate analyses?

Can subgroup analyses please be explored?

"Table 1 Characteristics of individuals from included records"

Can differences in sex ratio between the groups please be adjusted for as a potential confounder in the analyses?

Can additional demographics and characteristics please be reported and analysed in this study?

For instance, ethnicity and race, BMI, comorbidities, socioeconomic variables, and lifestyle behaviours?

"The pragmatic choice of using end of life as a reference in some of our analyses does not align with the view that ACP should not be limited to end-of-life communication. • The study incorporated national data in the analysis, though such data may not always be readily obtained in general practice settings. • The study population was relatively old due to matching with deceased individuals, which may limit generalizability to other populations aged 65 and over." [strengths and limitations]

and

"However, some limitations should be acknowledged. ACP is not defined as inclusive of end-of-life communication (7, 27) but we pragmatically choose end of life as a reference in our analyses. Further, we were able to incorporate national data to analyze referrals to the emergency department and hospital admissions, although such information may not be readily obtained in general practice data. Last, matching with the deceased individuals resulted in a relatively old study population and the prevalence of triggers found in this sample may not be generalizable to other populations of patients above age 65. However, we expect that triggers will be even less present in a younger control group."

A discussion on the study limitations is provided.

Thanks for providing a copy of the CODE-EHR checklist.

VERSION 1 - AUTHOR RESPONSE

Reviewer 1		
1. I have no additional comments	We thank the reviewer for taking time to review our manuscript.	
Reviewer 2		
1. This study builds on authors' previous work to establish a series of triggers and an optimum window for ACP discussion, by comparing the prevalence and effect of the triggers and how early the triggers appeared in the medical records with the optimal window. It utilizes a lot of real world data to further add to the model previously developed, however I have some questions as mentioned below.	We thank the reviewer for their assessment of our manuscript. It helped us greatly to improve our manuscript. We address the comments below.	
2. Although reference is made to the previous optimal window study, it might be helpful to describe what this means in terms of median days and window in Page 7 line 10 so that readers are familiar with the concept and its units. It is also a bit strange that given the variety of triggers and expected lifespan variation in the previous study, that a single window and mean could	In the revised manuscript we clarified the 'window of optimal ACP timing'.<br>In the previous study, our aim was to explore the optimal timing of ACP in individual records and to identify triggers that GPs recorded as indicators for initiating ACP. The optimal time window was identified by presenting anonymized routine electronic health records of patients who died from cancer,	Page 5, line 17-23, we added "The optimal time window had been identified in a previous study by presenting anonymized routine electronic health records of patients who died from cancer, organ failure, or multimorbidity to participating general practitioners (three records per GP). Participants were asked to determine the optimal moment to initiate

be calculated as the optimum- I would have expected that GPs in the previous study would have been more careful in saying there is no one size fits all for ACP?	organ failure, or multimorbidity to participating general practitioners (three records per GP). Participants were asked to determine the optimal moment to initiate advance care planning in individual records. We expressed the cumulative findings as the median number of days (with interquartile range) between the proposed time point and the patient's death across all reviewed records. Given indeed the wide variation in the optimal timing indicated by GPs in various health records based on various triggers, we interpreted the optimal timing to initiate ACP not as a fixed point but rather as a window of opportunity, with triggers serving as potential flags to indicate the start of this window.	advance care planning in individual health records. We then expressed the results as the median number of days (with interquartile range) between the proposed time point and the patient's death across all reviewed records (median optimal ACP timing 228 days before death, 25th – 75th percentile 65 - 458 days before death)."
3. For matching case-controls, is age alone enough for matching? Some justification should be given for using this as the matching criterion.	We matched on age, as there was a substantial initial difference between the case and control group, and age was considered an important factor likely to influence the prevalence of triggers. Additional matching criteria were deliberately avoided to maintain similarity with a population in general practice and thereby enhance the practical relevance of the findings.	Page 5, line 9-12, we changed "to minimize age related bias" to "We considered age the most important factor likely to influence the overall prevalence of triggers. We did not match on additional criteria for specific triggers, so as to preserve the population's similarity to a gp practice population."
4. From Table 1, it looks as if the case and control groups may have some baseline differences (ie the Living individuals appear to be younger in mean age, and more female). I wonder if these need to be controlled for in the regression. It might not change the outcomes, as the list of	The primary aim of our study was to describe the prevalence of triggers in real-life data and to explore their association with death and their relation to the optimal window. The reviewer is correct that the strength of association might not be entirely accurate. However, the observed	

triggers make sense as being linked to higher mortality, but the effect may not be as accurate if the baseline confounders are not controlled for.	associations are strong and based on a large dataset and we expect no major change after adjusting for sex. Moreover, age has been used for the matching and therefore there is no need to include this variable in the regression model. Including age again in the model would be redundant since the matching already controls for it.	
5. It is unclear how the list of triggers that were derived from the previous study as the list given in this study are very detailed and disease specific, whereas the reference does not have such a detailed list. I was curious about this, as some triggers were supposed to be associated with death (since they were included) but had an opposite outcome (e.g. osteoporosis and physiotherapy), so I was wondering how these outcomes were deemed to be ACP triggers, but could not find the details for this in this paper or the reference. Perhaps some explanation on how the list of triggers was discussed and derived would help.	We apologize for not clearly describing how we derived the list of triggers used in this study. The clinical indicators identified by GPs in the previous study were broader and based on qualitative data, whereas our current study required triggers based on coded information from primary care records (e.g. ICPC-codes). To make this translation, a group of three (one researcher involved in the analysis of previous study and two GPs from our team) translated the broad clinical indicators into more specific coded variables available in the database (see supplemental file). This process integrated the researcher's knowledge of the data from the previous study and the GPs' practical experience of how such information is usually recorded. In this way, we developed the more detailed, disease-specific list of triggers and both its development and analyses were part of the current study.	We clarified in the revised manuscript. On page 5-6, line 29-1, we added "WT (involved in the previous study's data analysis), PvP (GP and researcher) and MN (GP and researcher) translated the broad clinical indicators into more specific coded variables available in the primary healthcare data. This process integrated the researchers' knowledge of the clinical indicators from previous study with the GPs' practical experience of how such information is typically recorded in primary care."
6. As the window matching is based on a three year follow-up period (3 years before June 2018), some justification for this 3 year window should be given. It is quite striking that	In our previous study, we used a two-year window and observed that the vast majority of relevant triggers occurred well within this timeframe. For the current	Page 10, line 9-10, we added "Also, our time frame of identifying triggers up to three years before death captured a variety of relevant triggers"

the median days fall largely within the optimal window for the deceased cases in the majority of triggers and I question whether this might be due to the 3 year window- perhaps if the records were analyzed further back, the median days might go further back in terms of the trigger dates- for example medications might have been prescribed much earlier than the 3 years of the follow-up period. Some clarity on reasons for the 3 year window and how this might affect the median date of the trigger should be given.	study, we deliberately extended this to three years in order to assess whether triggers tend to occur within this "window of opportunity" or potentially earlier, and to ensure we did not miss such earlier signals.<br>Therefore the three-year window was a suitable choice. Although it is possible that certain triggers, such as medication prescriptions, may occur even earlier, we consider triggers which occur that much earlier of limited clinical value.	
7. For Table 1, how was Age in years calculated? At date of first and last record, or date of death? How does this fit into the 3 year window period for example.	We calculated age in years based on age at 01-01-2017 (page 5, line 3). For each individual, the follow-up period of three years varies relative to this date (page 6, line 5-8). For controls this date fell in the middle of their follow-up period. For deceased individuals, the follow-up periods ended at the date of death, so the position of 01-01-2017 within their individual follow-up period varied depending on when they died.	Page 7, table 1, we added "** calculated at 01-01-2017".
Reviewer 3		
1. This nested case-control study within a large dynamic population cohort dataset aims to explore whether routine electronic healthcare records can be used to identify triggers for initiating advance care planning (ACP) and the optimal time window to initiate ACP.	We thank the reviewer for thoroughly assessing our manuscript and for providing helpful suggestions to improve it.	
2. "Triggers most strongly associated with death (i.e. highest odds ratios) were consultations concerning malignancy, heart failure, dementia, general decline/feeling old and skin ulcers/pressure sores; opioid	We have included statistical outcomes in the results section in the abstract.	Page 2, we added ORs and 95% CIs in the results section and we re-ordered according to strength of the association.

prescriptions; emergency department referrals, registered home visits and hospital admissions. Those closest to the median of the optimal time window for ACP were consultations regarding dyspnea, general decline/feeling old, heart failure, skin ulcers/pressure sores and fever; opioid prescriptions; emergency department referrals, registered home visits and hospital admissions."<br>Can statistical outcomes please be reported alongside study findings in the Abstract?		
3. "Primary care population-based, anonymized data extracted from GP centers in the South-Holland province, The Netherlands...<br>We selected records of individuals aged ≥ 65 registered with their general practice from 01-01-2014 till 01-01-2017"<br>and<br>"We included 17098 included records, 4139 from deceased individuals (mean age 81) and 12.959 from living individuals (mean age 79)."<br>and<br>"This nested case-control study utilized Dutch routine primary health care data linked with national Statistics Netherlands data from the Extramural LUMC Academic Network (ELAN). "<br>Can the authors please further comment on whether the remaining analysed cohort can be considered to be demographically and characteristically representative for generalisation of the study outcomes?	We acknowledge that, due to the way we selected our cohort, it is not fully representative of the general primary care population aged >65. In particular, the mean age in our study (around 80 years) is relatively high. This should be considered when interpreting the findings, as noted in the limitations. The South Holland region includes both urban and rural areas. Requiring patients to be in the database for at least three years before follow-up, we have selected a group with consistent registration in a GP practice in the ELAN network, but this was necessary to ensure sufficient longitudinal information prior to death of the deceased individuals. Taken together, we believe our cohort still provides a reasonably representative sample of the older primary care population.	

4. "Cases were individuals who died between 01-01-2017 and 01-01-2020. Controls were individuals who remained alive"<br>Can the authors please clarify how accidental (e.g. non-clinical, such as a car accident) deaths are accounted for in the analyses?	Cases were defined as all individuals who died between 01-01-2017 and 01-01-2020, irrespective of cause of death. We did not exclude accidental deaths, as we considered it important that the study population reflects the real-life general population. Given the low proportion of accidental deaths in this age group, we do not expect this to have materially affected our findings.	Page 5, line 5, we added "(both non-accidental and accidental deaths)"
5. "Cases were matched by age to controls in a 1:4 ratio" [Abstract]<br>and<br>"we matched the deceased individuals (cases) to living individuals (controls) by age at a 1:4 ratio to minimize age-related bias (figure 1)"<br>Can the matching algorithm or process please be specified in more detail?	We apologize we did not specify the method used for matching. We now specified this in the method section of the manuscript.	Page 5, line 8-9, we added "using propensity score matching, applying nearest-neighbour matching with a 0.2 SD caliper"
6. Can the authors please consider also matching on other factors, such as sex?	We matched on age, as there was a substantial initial difference between the case and control group, and age was considered an important factor likely to influence the overall prevalence of triggers. We purposefully refrained from additional matching criteria to maintain similarity with a gp practice population and thereby enhance the practical relevance of the findings.<br>We also consider sex unlikely to exert a consistent effect across all triggers, given their heterogeneity and wide variation, and therefore did not include it as a matching criterion.	Page 5, line 9-11, we changed "to minimize age related bias" to "We considered age the most important factor likely to influence the overall prevalence of triggers. We did not match on additional criteria for specific triggers, so as to preserve the population's similarity to a gp practice population."
7. "The study period was chosen to select data collected before the COVID-19 pandemic, as this might have	We believe that the COVID-19 pandemic altered healthcare seeking behavior and GP consultation patterns in the	Page 10, line 17-20, we added "Additionally, we acknowledge that the manner and extent of registration may have evolved

affected the consultation behavior of patients and the registration by GPs of data and thus the prevalence of ACP triggers."<br>Can the authors please comment on whether they consider study outcomes to be applicable today (i.e. post COVID-19 pandemic)?	first few years, that's why we chose not to use data of this period. We do not, however, expect that these changes have persisted in the post-pandemic era we are in now. We acknowledge that the manner and extent of registration may have evolved over time, as registration has become increasingly emphasized in recent years. This could potentially lead to some differences in outcomes between our study period and the present. Nonetheless, we consider it likely that the key triggers we identified as important and strongly associated with the outcome remain relevant today. Therefore, we believe our findings continue to be applicable to current primary care populations.	between our study period (2017-2020) and today, which could potentially result in differences in outcomes. Nonetheless, the key triggers remain relevant to current practice."
8. "From primary healthcare data, we extracted consultation registrations according to the WHO International Classification of Primary Care (ICPC)(24), laboratory test results, registered correspondence with paramedics, and prescribed medication according to the Anatomical Therapeutic Chemical (ATC) (25). From the Statistics Netherlands data, we extracted referral to the emergency department- and hospital admission data. We extracted all data covering three years for everyone; three years preceding death for deceased individuals, and three years preceding 1st of June 2018 (midway the follow-up period) for individuals who were alive at the end of the follow-up period (figure 1). Data linkage was performed	The data included in our study were complete electronic patient files. For the primary care data (consultation registrations, laboratory results, correspondence and prescribed medication), completeness depends on what and how GPs record information; as with all routine healthcare data, underreporting or missing entries is possible. However, any future tool or algorithm would also need to rely on real-life data which is subject to missing data and registration bias. We minimized missing data regarding date of birth, information on death and referral- and hospital admission data by including Statistics Netherlands data which is complete and reliable.	Page 10, line 7-9, we changed "and we utilized real-life data, ensuring applicability in clinical practice" to "By using real-life data, we ensured that our findings are applicable in clinical practice, where identification tools ultimately need to be implemented."<br><br>Page 10, line 13-15, we added "Further, our study relies on routinely recorded primary care data and coded data only, which may result in underreporting or missing entries. We mitigated part of this issue by incorporating national data of Statistics Netherlands."

using record identification numbers. Data was selected, operationalized (Supplemental file, table S1 and table S2), linked, and analyzed within a protected remote access environment of Statistics Netherlands"<br>Can the authors please confirm if there was any missing data?		
9. "A univariable logistic regression model was estimated to study the association of a triggers' presence with death; odds ratios along with 95% confidence are reported. Additionally, for triggers with an odds ratio of 1.5 or higher (26), we described the timing of the triggers (days before death, median and 25th-75th percentile) in the records of deceased individuals relative to the 'window of optimal ACP timing' from our previous study"<br>Can the authors please also conduct multivariate analyses?	We appreciate the suggestion of the reviewer to conduct multivariate analyses. Our study was descriptive in nature, aiming to explore whether the single specific triggers could be identified in the records and are associated with death and could potentially serve as predictors for future algorithms. Multivariate analyses (multivariable, with multiple independent factors) are typically used to adjust for confounders when assessing causal relationships, or to develop prognostic models. We believe that translating the triggers for ACP identified previously to real-world codes and chart data, univariate (bivariable) associations represent the necessary first step and addresses the objectives of this study.	
10. Can subgroup analyses please be explored?	We appreciate the reviewers suggestion. However, we think subgroup analyses should be avoided because there is the risk of generating false-positive results from multiple comparisons and false-negative results due to insufficient statistical power in smaller groups. This could potentially lead to incorrect conclusions about the effect of covariates.	

11. "Table 1 Characteristics of individuals from included records"<br>Can differences in sex ratio between the groups please be adjusted for as a potential confounder in the analyses?	We acknowledge the reviewer's point regarding baseline differences in sex ratio. Including sex as a covariate in the model will provide adjusted odds ratios for the association between triggers and death. This adjustment may slightly attenuate the associations between the independent variables and the outcome; however, given the strength of the associations observed for each trigger, no major changes and no impact on clinical relevance is expected.	
12. Can additional demographics and characteristics please be reported and analysed in this study?<br>For instance, ethnicity and race, BMI, comorbidities, socioeconomic variables, and lifestyle behaviours?	These data were not available to us, as they are not routinely or reliably recorded in the primary healthcare database, especially not in coded form. Considering the aim of our study—to provide a descriptive, real-life overview of primary care data—and to maintain practical relevance, we considered inclusion of these variables beyond the scope of this first-step exploratory study.	
13. "The pragmatic choice of using end of life as a reference in some of our analyses does not align with the view that ACP should not be limited to end-of-life communication. • The study incorporated national data in the analysis, though such data may not always be readily obtained in general practice settings. • The study population was relatively old due to matching with deceased individuals, which may limit generalizability to other populations aged 65 and over." [strengths and limitations]<br>and<br>"However, some limitations	Indeed, our study has limitations, which we have expanded in response to the reviewers' comments. At the same time, we made pragmatic methodological choices to keep out study closely aligned with clinical practice, thereby supporting the potential future implementation of tools identifying the need for ACP.	

should be acknowledged. ACP is not defined as inclusive of end-of-life communication (7, 27) but we pragmatically choose end of life as a reference in our analyses. Further, we were able to incorporate national data to analyze referrals to the emergency department and hospital admissions, although such information may not be readily obtained in general practice data. Last, matching with the deceased individuals resulted in a relatively old study population and the prevalence of triggers found in this sample may not be generalizable to other populations of patients above age 65. However, we expect that triggers will be even less present in a younger control group."<br>A discussion on the study limitations is provided.		

VERSION 2 - REVIEW

**Reviewer**	**3**
**Name**	**Kelson, Zoe**
**Affiliation**	**University of Exeter, Mathematics**
**Date**	**14-Oct-2025**
**COI**	

Thanks to the authors for the responses and revisions.

Author response:

"We acknowledge that, due to the way we selected our cohort, it is not fully representative of the general primary care population aged >65. In particular, the mean age in our study (around 80 years) is relatively high. This should be considered when interpreting the findings, as noted in the limitations. The South Holland region includes both urban and rural areas. Requiring patients to be in the database for at least three years before follow-up, we

have selected a group with consistent registration in a GP practice in the ELAN network, but this was necessary to ensure sufficient longitudinal information prior to death of the deceased individuals. Taken together, we believe our

cohort still provides a reasonably representative sample of the older primary care population."

Reviewer request:

Can this response to reviewer please be further incorporated into the article?

Author response:

"Cases were defined as all individuals who died between 01-01-2017 and 01-01-2020, irrespective of cause of death. We did not exclude accidental deaths, as we considered it important that the study population reflects the real-life general population. Given the low proportion of accidental deaths in this age group, we do not expect this to have materially affected our findings."

Reviewer request:

Can this please be further detailed in the article, specifying the low proportion of accidental deaths in this age group?

Author response and amendment:

"Page 5, line 8-9, we added “using propensity score matching, applying nearest-neighbour matching with a 0.2 SD caliper”"

Reviewer request:

The authors have conducted a rigorous matching methodology.

Can this please be described in full, as well as showing standardised mean differences (SMDs) pre and post matching?

Author response:

"We acknowledge the reviewer’s point regarding baseline differences in sex ratio. Including sex as a covariate in the model will provide adjusted odds ratios for the association between triggers and death. This adjustment may slightly attenuate the associations between the independent variables and the outcome; however, given the strength of the associations observed for each trigger, no major changes and no impact on clinical relevance is expected."

Reviewer request:

Can the authors please adjust for sex in a sensitivity analysis?

VERSION 2 - AUTHOR RESPONSE

Comments/requests	Response	Change in revised manuscript (if any)<br>(Page and line numbers are the numbers in the revised manuscript with track changes)
Author response:<br>"We acknowledge that, due to the way we selected our cohort, it is not fully representative of the general primary care population aged >65. In particular, the mean age in our study (around 80 years) is relatively high. This should be considered when interpreting the findings, as noted in the limitations. The South Holland region includes both urban and rural areas. Requiring patients to be in the database for at least three years before follow-up, we have selected a group with consistent registration in a GP practice in the ELAN network, but this was necessary to ensure sufficient longitudinal information prior to death of the deceased individuals. Taken together, we believe our<br>cohort still provides a reasonably representative sample of the older primary care population."<br>Reviewer request:<br>Can this response to reviewer please be further incorporated into the article?	We have incorporated our previous response in the revised manuscript.	We added on page 10 line 30-31 and page 11 line 1-2, 'Last, our cohort of urban and rural areas in South Holland with a mean age of around 80 may not be fully representative of the countries' older primary care population.'<br>And 'Moreover, we have selected a group of patients who remained in a single GP practice for the whole study period to ensure sufficient longitudinal follow-up.'
Author response:<br>"Cases were defined as all individuals who died between 01-01-2017 and 01-01-2020, irrespective of cause of death. We did not exclude accidental deaths, as we considered it important that the study population reflects the real-life general population. Given the low proportion of accidental deaths in this age group, we do	We have incorporated our previous response in the methods section with a specification of the low proportion of accidental death, together with a reference.	In the methods section, on page 5, line 20-23 we added "We did not exclude accidental deaths, as we aimed for the study population to reflect the real-world general population. Regardless, given the low proportion of accidental deaths among the older population (<0.5% in 2017, 2018 and 2019), we do not expect their inclusion

not expect this to have materially affected our findings."<br>Reviewer request:<br>Can this please be further detailed in the article, specifying the low proportion of accidental deaths in this age group?		to have a material impact on findings."<br><br>Added reference:<br>https://www.cbs.nl/nl-nl/longread/statistische-trends/2025/trends-in-sterfte-en-doodsoorzaken-2014-2024?onepage=true#c-4--Doodsoorzaken--absoluut--relatief-en-gestandaardiseerd
Author response and amendment:<br>"Page 5, line 8-9, we added "using propensity score matching, applying nearest-neighbour matching with a 0.2 SD caliper""<br>Reviewer request:<br>The authors have conducted a rigorous matching methodology.<br>Can this please be described in full, as well as showing standardised mean differences (SMDs) pre and post matching?	In the revised manuscript we described the used method more extensive and reported SMD's pre- and post-matching.	In the methods section, page 5, line 9-14, we added "This method pairs each case with the nearest control within the specified caliper range (0.2 SD), ensuring matched individuals are similar in their propensity scores and thus sufficiently similar in age for our descriptive study. Despite a reduction of the standardized mean difference (SMD) from 1.079 pre-matching to 0.348 post matching, the desirable SMD of <0.1 was not achieved. However, we prioritized retaining the larger sample size."
Author response:<br>"We acknowledge the reviewer's point regarding baseline differences in sex ratio. Including sex as a covariate in the model will provide adjusted odds ratios for the association between triggers and death. This adjustment may slightly attenuate the associations between the independent variables and the outcome; however, given the strength of the associations observed for each trigger, no major changes and no impact on clinical relevance is expected."<br>Reviewer request:<br>Can the authors please adjust for sex in a sensitivity analysis?	We purposefully refrained from adjustments as the aim of our study was to describe the prevalence of triggers in real-life data and to explore their association with death and their relation to the optimal window. We did not aim to develop a prediction or causal model. We decided to adjust for age in our samples of older people because the comparative cohort was younger due to age affecting the overall prevalence of triggers of ACP and affecting mortality. Adjustment for any other factor regardless of this being selected demographic or other factors is expected to affect	

	triggers differentially and is inconsistent with our descriptive goal. Adjustment would complicate the interpretation of usefulness of single triggers for identifying patient who might benefit from ACP in general practice.	
